# Streamlined analysis of duplex sequencing data with Du Novo

**DOI:** 10.1186/s13059-016-1039-4

**Published:** 2016-08-26

**Authors:** Nicholas Stoler, Barbara Arbeithuber, Wilfried Guiblet, Kateryna D. Makova, Anton Nekrutenko

**Affiliations:** 1 Graduate Program in Bioinformatics and Genomics, The Huck Institutes for the Life Sciences, Penn State University, 505 Wartik Lab, University Park, PA 16802 USA; 2 Institute of Biophysics, Johannes Kepler University, Linz, Austria; 3 Department of Biology, Penn State University, 310 Wartik Lab, University Park, PA 16802 USA; 4 Department of Biochemistry and Molecular Biology, Penn State University, University Park, PA USA

**Keywords:** Duplex sequencing, Low frequency polymorphism discovery, Next generation sequencing, Genomic data analysis

## Abstract

Duplex sequencing was originally developed to detect rare nucleotide polymorphisms normally obscured by the noise of high-throughput sequencing. Here we describe a new, streamlined, reference-free approach for the analysis of duplex sequencing data. We show the approach performs well on simulated data and precisely reproduces previously published results and apply it to a newly produced dataset, enabling us to type low-frequency variants in human mitochondrial DNA. Finally, we provide all necessary tools as stand-alone components as well as integrate them into the Galaxy platform. All analyses performed in this manuscript can be repeated exactly as described at http://usegalaxy.org/duplex.

**Electronic supplementary material:**

The online version of this article (doi:10.1186/s13059-016-1039-4) contains supplementary material, which is available to authorized users.

## Background

The term “genetic variation” is often used to imply allelic combinatorics within a diploid organism such as humans or *Drosophila*. Yet the majority of organisms in the biosphere are not diploid (prokaryotes and viruses), and even those that are include non-diploid genomes such as mitochondria and chloroplasts. Identification of genetic variants—e.g., single nucleotide polymorphisms (SNPs) and small indels—is especially challenging in non-diploid systems due to the lack of a simple “homozygote-or-heterozygote” expectation: a heterozygous site may have not just two but multiple allelic variants, with frequencies ranging anywhere from 0 to 1 [1, 2]. Because high-throughput sequencing technologies exhibit considerable amounts of noise [3], it becomes increasingly difficult to reliably call variants with frequencies below 1 % [4–9]. In these situations increased sequencing depth does not improve the predictive power but instead introduces additional noise. This complicates the identification of low-frequency variants that is becoming critically important in a variety of applications. For example, humans have numerous disease-causing mitochondrial variants where the disorder penetrance is proportional to the allele frequency [10]. Because dramatic shifts in allele frequency can occur during mitochondrial bottleneck during oogenesis, a disease-causing variant present at a very low frequency in the mother may increase in frequency in the child to exhibit a disease phenotype. The lack of cures for diseases caused by mitochondrial DNA mutations and the recent regulatory approval of tri-parental in vitro fertilization by the UK House of Commons makes it critical to identify low-frequency variants in the human mitochondrial genome [11]. Other examples illustrating the importance of discovering low-frequency genome alterations include tracking mutational dynamics in viral genomes, malignant lesions, and somatic tissues [12, 13].

**Fig. 1:**
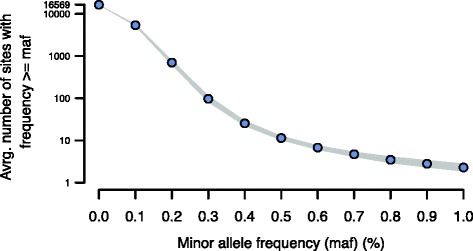
The relationship between the minor allele frequency (*maf*) threshold (*x-axis*) and the total number of variable sites (*y-axis*) detected by [7]. Lowering the MAF threshold leads to an exponential increase in the number of variable positions. The image was generated by applying variable MAF thresholds to data from 156 human samples and plotting the average number of variable sites at a given MAF threshold. The *line thickness* corresponds to the 95 % confidence interval around the mean value

Today the vast majority of strategies for the identification of low-frequency sequence variants rely on next-generation sequencing technologies. Noise reduction in these approaches ranges from simple base-quality filtering to complex statistical strategies incorporating instrument and mapping errors [4, 7, 14]. However, there is still considerable uncertainty about alternative alleles with frequencies below 1 %. For example, Fig. 1 illustrates the number of potential polymorphisms observed within the human mitochondrial genome as a function of the allele frequency cutoff. At 1 % there is an average of three sites [7], while at 0.75 % the number surpasses 10, and, finally, around 0.1 % almost all sites appear polymorphic. Clearly, the majority of these sites are false-positives but how does one know for certain? Potentially, highly sensitive techniques with a high dynamic range, such as droplet digital PCR [15, 16], can be used to validate each site, but it would quickly become prohibitively expensive and laborious to perform this on hundreds or thousands of sites.

An approach that offers a potential solution is duplex sequencing [17]. This recently developed method was designed to increase sequencing accuracy by over four orders of magnitude. Duplex sequencing uses randomly generated barcodes to uniquely tag each molecule in a sample. The tagged fragments are then PCR amplified prior to the preparation of a sequencing library, creating fragment families characterized by unique combinations of barcodes at both 5′ and 3′ ends (a conceptually similar primer ID approach [12] allows tagging of cDNA fragments at the 5′ end only). A family contains multiple reads, each originating from a single input DNA fragment. A legitimate sequence variant will thus be present in all reads within a family. In contrast, sequencing and amplification errors will manifest themselves as “polymorphisms” within a family and so can be identified and removed. A consensus can be called from these read families. The consensus of all the reads originating from the same strand reduces errors originating from sequencing and PCR amplification. Then, comparing consensus sequences from complementary strands can identify early PCR errors.

Despite the fact that duplex sequencing promises great advances, the methods for both experimental and computational aspects of this technique are still evolving. In fact, the latter is lagging as it is based on alignment to a reference genome, which is disadvantageous for several reasons. The use of a reference genome biases results toward that reference, affecting studies using de novo assembly or studies examining indels or other alleles that diverge far enough from the reference to cause alignment difficulties. The current analysis method also removes a large (and potentially useful) fraction of the original data due to stringent filters and uses suboptimal tools for variant identification. Here we describe an alternative analysis strategy which removes reliance on a reference sequence, preserves a higher proportion of the input reads, and can be deployed as a standalone application or as a part of the Galaxy system. We demonstrate the application of this approach by validating rare variants in the human mitochondrial genome.

## Results and discussion

### A reference-free approach

**Fig. 2:**
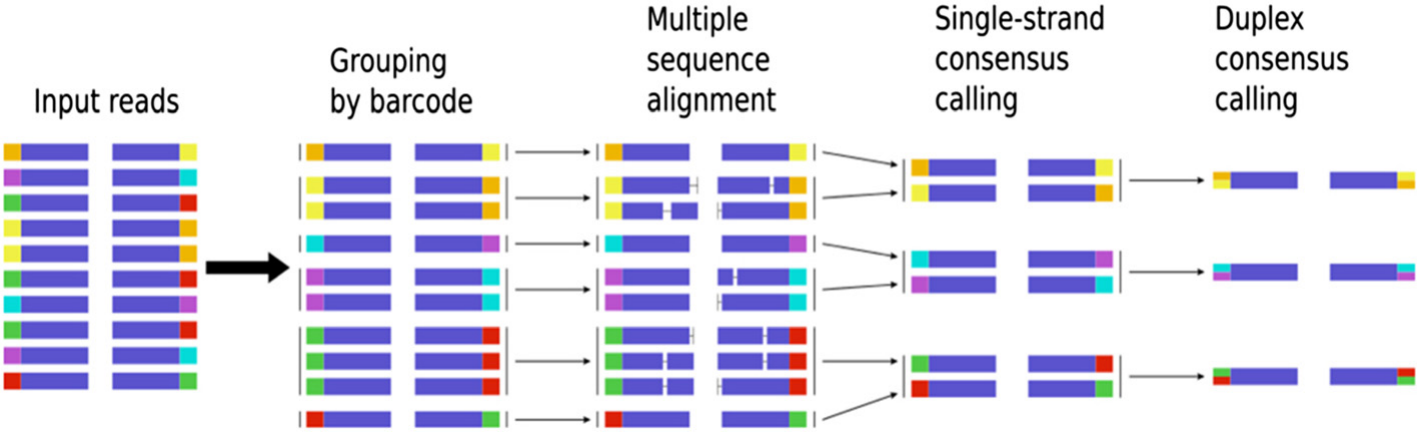
The Du Novo approach. First, reads tagged with identical barcodes are grouped into strand-specific families. Reads within each family are aligned and single-stranded consensus sequences (SSCSs) are generated. Finally, the SSCSs are reduced into duplex consensus sequences (DCSs)

Our approach is outlined in Fig. 2. First, paired reads generated from a duplex sequencing experiment are merged into families. This is performed by sorting according to the barcode. Each fragment is expected to be represented by two single-stranded families corresponding to each strand. These two single-stranded families are expected to have the same unique tags but in the opposite order: the α tag from one single-stranded family will be the β tag in the other (also see Fig. 1 in [17]). In order to group single-stranded families from the same fragment together, we normalize the order of the concatenation to produce a “canonical barcode” (a concatenated string consisting of α and β tags), which will be identical for both strands. The order of the canonical barcode is determined by a simple string comparison. Sorting the output groups the reads so that the two families constituting each duplex will be adjacent, with the read pairs separated by strand.

Next, the reads in each single-stranded family are aligned to themselves and these alignments are used to call the single-stranded consensus sequence (SSCS). First, a threshold is applied, requiring a user-specified number of reads to produce a consensus (three by default). The consensus calling is conducted by determining the majority base at each position. If no base is in the majority, “*N*” is used. Positions with gaps are considered in the same way as bases. Quality filtering is performed at this stage: bases with a Phred quality score [18] lower than a user-specified threshold are not counted (20 is used by default). For positions with gaps, a quality score is calculated by considering the qualities of eight neighboring bases. The calculated score is a weighted average, with the weight decreasing linearly with distance from the gap. Finally, a duplex consensus is called using the two SSCSs. The SSCSs are aligned using the Smith–Waterman algorithm [19] and then each pair of bases is compared. If the bases agree, that base is used in that position to generate duplex consensus. If they disagree, the International Union of Pure and Applied Chemistry (IUPAC) ambiguity code for the two bases is used. Gap and non-gap characters produce an “*N*”. In the end the above approach reduces the initial set of sequencing reads to a collection of duplex consensus sequences (DCSs; as the duplex sequencing experiments are performed with paired-end reads, the output of the procedure also consists of pairs corresponding to forward and reverse double-stranded consensuses). DCSs are then filtered (i.e., sequences with ambiguous nucleotides can be removed or trimmed), mapped against the reference genome, and realigned to normalize gap-containing regions and the resulting alignments are used to call variants. In this scenario variants are expected to have the full spectrum of allele frequencies between 0 and 1 and do not follow a diploid expectation. For that reason we use variant callers capable of dealing with this limitation such as the Naive Variant Caller (NVC) [20] or Freebayes [21]. Finally, variant calls are post-processed to compute the strand bias (using formulae from Guo et al. [22]). This approach is implemented in a pipeline relying exclusively on open-source software (https://github.com/galaxyproject/dunovo and accessible through the Galaxy system). We termed this approach Du Novo—for duplex sequencing de novo assembly-based calling.

### Du Novo reliably identifies very low frequency variants

First, we evaluated the performance of Du Novo by applying it to a dataset generated from a simulated mixing experiment. The advantage of performing the simulation is that the “truth” is known explicitly. We randomly generated 21 “heteroplasmies” by modifying human mitochondrial sequence. This altered version of the mitochondrial genome was then “mixed” with unmodified reference sequence at a ratio of 1:10,000 (thus, each “heteroplasmy” in this mix has the frequency of 0.0001) and a duplex experiment was simulated on the mixture. This was done by randomly generating 2500 fragments from the altered sequence and 25,000,000 fragments from unmodified reference, adding barcodes, and performing in silico PCR and sequencing (see “Methods”). The polymerase error rate in PCR and sequencing was set at 0.1 % per base. After applying Du Novo to the simulated reads and aligning the DCSs to the mitochondrial reference, the median read depth was 166,574×. Next, we identified all variable sites and filtered them using a series of minor allele frequency (MAF) thresholds and requiring a minimum DCS coverage of 10,000. The relationship between MAF thresholds and the numbers of false positives and false negatives is shown in Additional file 1: Figure S1. Du Novo correctly identifies 20 of the 21 variants with no false positives. The remaining variant was present at a frequency of 0.00004 (likely a result of random fluctuation), along with 46 false positives with an equal or higher MAF.

### Comparison with original approach: Du Novo replicates published estimates

To assess the performance of our method on real-world data and to compare it head-to-head with the original approach of Kennedy et al. [23], we re-analyzed a recently published dataset by Schmitt and colleagues [13] using both methods. In [13] the authors employed duplex sequencing to identify a rare mutation at the *ABL1* locus responsible for resistance to the chronic myeloid leukemia therapeutic compound imatinib. The resistance is conferred by the presence of G-to-A substitutions within the *ABL1* coding region resulting in an E279K amino acid replacement. This substitution is present in a small sub-clonal subset of cells at an ~1 % frequency. The dataset (Sequence Read Archive accession SRR1799908) contains 6,921,891 read pairs representing 1,468,089 unique tag combinations (potential families; Table 1).

**Table 1:** Characteristics of *ABL1* and SC8 duplex sequencing experiments

Number of	*ABL1*	SC8
Read pairs	6,921,891	17,385,100
Unique tags	1,467,768	2,100,705
Unique αβ configurations	748,411	1,148,444
Unique αβ configurations with 1 read pair	677,069	884,295
Unique αβ configurations with ≥3 read pairs	60,333	222,823
Unique βα configurations	743,669	1,092,748
Unique βα configurations with 1 read pair	672,946	832,875
Unique βα configurations with ≥3 read pairs	60,032	140,486
Unique αββα	24,313	140,485
Unique αββα with ≥3 read pairs on both strands	20,746	109,999
Reads within αββα families with ≥3 read pairs on both strands	2,156,105	8,636,692

First, we analyzed this dataset with Du Novo. Requiring each family to contain at least three reads reduced this number to 120,365 SSCSs and reconciling these into DCSs further reduced this number to 20,746 DCSs constructed from 2,083,140 read pairs (the remaining 6,921,891 − 2,083,140 = 4,838,751 were represented by families with less than three reads and were omitted; see Additional file 2: Figure S2). Mapping DCSs to the reference human genome showed the G-to-A substitution with frequency varying from 1.28 to 1.31 % depending on the variant caller (NVC [20] and FreeBayes [21], respectively) but irrespective of the mapper used (BWA-MEM [24] or BWA [25]).

**Fig. 3:**
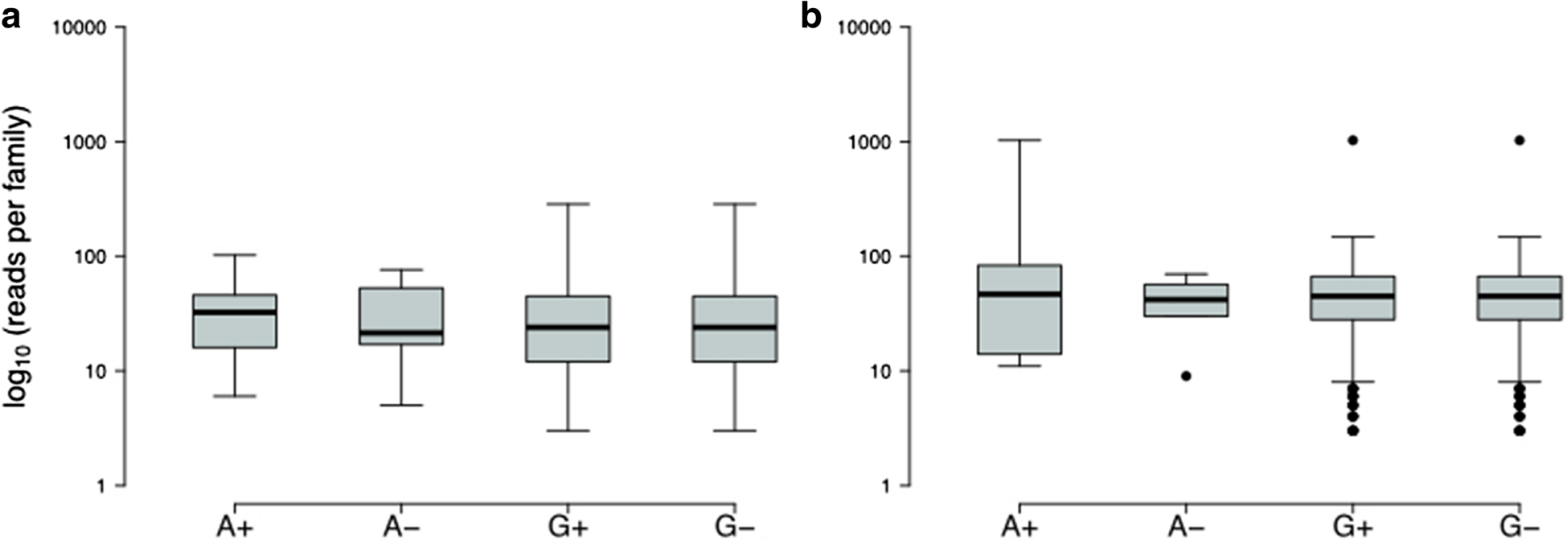
Distribution of family sizes (number of reads per family) supporting A and G alleles on both strands (plus and minus) for **a** site 130,872,141 in the *ABL1* dataset and **b** site 13,708 in the SC8 dataset

Next, we repeated this experiment with the published duplex sequencing pipeline [23]. This produced 1.29 and 1.31 % frequencies at the G-to-A substitution site for NVC and FreeBayes, respectively. Thus, the allele frequency estimates were essentially identical between the two approaches. Du Novo produced a higher depth at the variable site: 1099 for our method versus 618 for the published pipeline [23]. However, at such low allele frequencies even formidable coverage results in a relatively small proportion of reads supporting the minor allele. For example, in the case of this analysis the minor allele (“A”) is supported by 14 duplex consensuses from the total of 1099, resulting in a MAF of 1.28 %. Yet each of these 14 families is in turn derived from multiple starting reads ranging from a minimum of 5 to a maximum of 102 (Fig. 3a), providing additional support for the reliability of the minor allele calls.

### Using Du Novo to call low-frequency heteroplasmies at mitochondrial DNA

After ensuring the adequate performance of Du Novo on the *ABL1* data, we applied it to the identification of low-frequency variants in human mitochondrial DNA (mtDNA). Previously, we have reported 174 point heteroplasmies identified from the analysis of mtDNA in 39 mother–child pairs (a total of 156 samples = 39 mothers × 2 tissues + 39 children × 2 tissues [7]). We chose family SC8 as it displays significant variability across samples and individuals. This family contains two heteroplasmic sites—at positions 7607 and 13,708. According to our published results [7], the MAFs at site 7607 are 0.7, 1.1, 0.0, and 0.0 % in mother’s buccal tissue, mother’s blood, child’s buccal tissue, and child’s blood, respectively. The corresponding MAFs at site 13,708 are 0.0, 0.0, 2.2, and 1.6. To verify these frequencies, we performed the duplex sequencing experiment using genomic DNA extracted from SC8 child buccal tissue in which mtDNA has been enriched via long-range PCR as previously described in [7]. We started with 17,385,100 read pairs that contained 2,100,704 unique tags and were assembled into 82,230 DCSs. The estimated allele frequency at position 13,708 was 0.53 %, a figure substantially lower than the 2.2 % estimated previously [7]. The coverage at this site was 1138 with six reads representing the minor allele (“A”). To check the reliability of this call we estimated strand bias (SB; using formula 1 from [22]) for all sites with MAF ≥0.5 %. There were 20 sites (excluding 13,708) with MAF ranging from 0.51 to 21.2 % and with SB values ranging from 0.94 to 6.08 (the lower the value, the less SB there is at a site; 0 is an ideal value [22]). SB = 0.01 at site 13,708, which is outside of the SB distribution for all other variable sites in our sample, strongly suggesting that this is the only true heteroplasmy in this sample. In addition, examining individual DCSs at this site indicates that each of them is generated from a large number of original reads (Fig. 3b) confirming this polymorphism, albeit at a significantly lower frequency.

### The utility of SSCSs

In the SC8 experiment described above, we estimated the MAF at site 13,708 to be 0.53 %—a much lower value compared with the original one (2.2 %) obtained from re-sequencing [7]. The likely cause of this deviation lies in the design of the duplex experiment. In this study we performed duplex sequencing not directly on mtDNA but instead on products of a long-range PCR (see “Methods”). In this particular case this is unavoidable as the samples are obtained by a minimally invasive “cheek swab”, resulting in a very low concentration of mtDNA. The core issue is that complementary strands of the resulting PCR products (the starting material for our duplex sequencing experiment) can randomly pair after amplification, forming heteroduplexes and leading to an underestimation of MAFs when using DCSs only (Additional file 3: Figure S3). To test whether this indeed is the cause of MAF underestimation, we performed variant calling using SSCSs instead of DCSs and obtained a MAF of 1.7 % (strand bias = 0.02 and depth = 4548), a value much closer to the 2.2 % reported in the original publication. Thus, although the background error frequency is higher for SSCSs in comparison with DCSs [17] in certain situations, such as experiments using ampliconic DNA, the use of SSCSs for polymorphism detection may be preferable to obtain more accurate allele frequencies.

### Loss of data as a result of sequencing errors in duplex tags

One of the fundamental weaknesses of duplex sequencing is the fact that the majority of families in a duplex experiment contain only a single read pair (Additional file 4: Figure S4). This eliminates a substantial amount of otherwise useful data from the analysis, contributing to the inefficiency of the current protocol. To understand the potential sources of read loss, we examined individual stages of the duplex analysis process. This information is compiled in Table 1 and is based on the re-analysis of both previously published data (*ABL1* data) [13] and results generated in our laboratory (the SC8 dataset described above). Both cases feature a large number of initial read pairs and unique tags. However, these numbers are rapidly reduced by requiring at least three reads within each single-stranded family. Combining SSCSs into DCSs also greatly reduces the number of useful sequences since both strands must be present and meet the three-read threshold. One potential explanation for the large number of families with only one read pair is sequencing errors within duplex tags. Each barcode with an error will almost certainly be unique, creating an entirely new apparent family with only one member. The number of reads with an erroneous barcode may be a minority but this can still result in the number of families with erroneous barcodes being very high (a majority). The fraction of erroneous barcodes (*r*) can be expressed in the following form:

1 $$ r = 1\hbox{--} {\left(1\ \hbox{--} E\right)}^l $$

where *E* is the per-base error rate and *l* is the barcode length (in this case 24 as it is a combination of α and β tags, each of which is 12 nucleotides). Here, *E* is a cumulative error rate taking into account the chance of a mutation at every cycle of PCR plus the sequencing reaction. The cumulative error rate can be calculated from the error rate at each stage using the same equation (Eq. 1), this time using *E* as the error rate per base per stage, *l* as the number of stages (number of PCR cycles plus 1 for the sequencing reaction), and *r* as the cumulative error rate. Even assuming a low per-stage error rate of 0.1 %, this gives a cumulative error rate of about 3 %. Using this in Eq. 1 again, we obtain the fraction of barcodes expected to contain an error to be 52.5 %:

2 $$ r = 1\hbox{--} {\left(1\hbox{--} 0.03\right)}^{24} \approx 0.525 $$

Now, suppose in a hypothetical duplex experiment ten initial fragments of DNA were ligated with α and β adapters (a unique α and β for each of the ten fragments) and the subsequent PCR amplification and Illumina sequencing process produced 100 read pairs (10 pairs per original fragment). If there are no errors, these 100 read pairs should be recognized as members of ten duplex families during the analysis stage. If we now factor in the erroneous barcode rate of ~52 % calculated above, one would observe 62 total families: ten real families and 52 artifactual families consisting of a single read pair. This phenomenon increases the total number of families by reducing the read count within legitimate families—a trend apparent in real data (Additional file 2: Figure S2). Furthermore, the relationship between the number of single-read families and the total number of reads can serve as a proxy for the error rate. For example, in the SC8 experiment there were 1,717,170 single read families and 17,385,100 total read pairs. Assuming that all single read families are byproducts of sequencing errors within duplex tags, this gives 1,717,170/17,385,100 = 0.098 as the fraction of erroneous barcodes (*r*). With *l* = 24 we can solve Eq. 1 for *E* obtaining an estimate of ~0.4 % for the cumulative error rate.

To test this reasoning we simulated duplex experiments with different error rates. The starting distribution of family sizes was constant in each case, with 1.20 % of fragments producing a family with only one read. With an error rate of zero, the proportion of output families which were composed of a single read was, as expected, precisely 1.20 % (Additional file 4: Figure S4), meaning no excess beyond those with a natural family size of one. When the error rate was raised to 0.1 % per base per cycle of PCR/sequencing reaction, 75.5 % of output families were composed of a single read. This meant that 74.3 % of families were artifacts consisting of a read originating from a fragment that produced multiple reads. Instead of being grouped with its sibling reads, each of these instead was grouped by itself because of an error in the barcode.

While this was only a simulation and the above calculations make a number of simplifying assumptions, they nevertheless highlight the significance of sequencing errors within tags as one of the main causes of data loss. We are currently developing a family reconstruction approach that would allow mismatches in tags and is expected to significantly reduce the number of single read families.

### Interactive analysis of duplex data

**Fig. 4:**
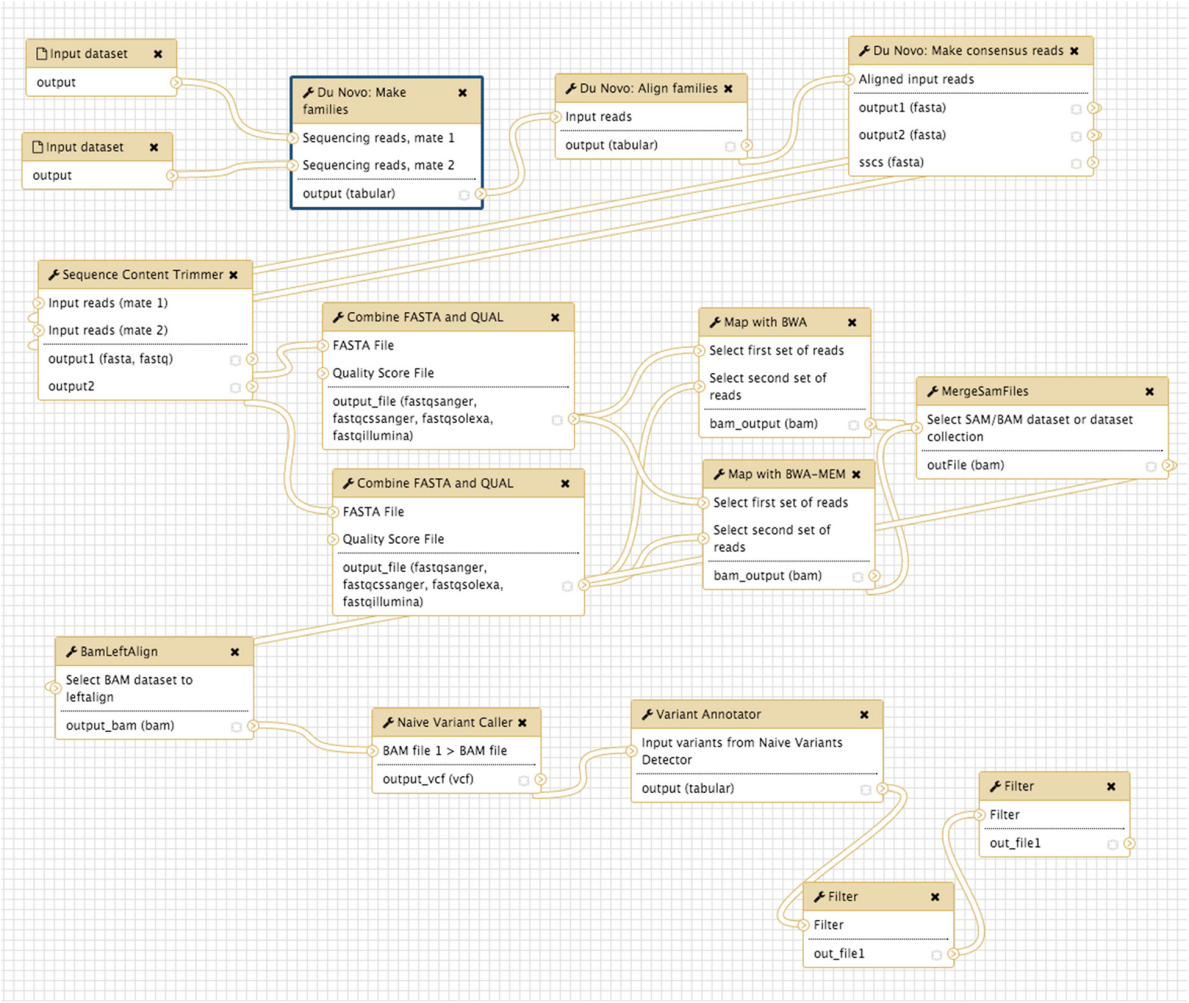
A complete workflow implementing the Du Novo approach to variant discovery from duplex sequence data. It is accessible from http://usegalaxy.org/duplex

The underlying components of the Du Novo process are distributed as an open source software and can be used from the command line (https://github.com/galaxyproject/dunovo). However, to increase the number of potential users we also make Du Novo accessible through the Galaxy system (http://usegalaxy.org). Figure 4 illustrates all stages of the duplex analysis workflow. This example begins with fastq datasets generated by an Illumina machine that are used as inputs in the Du Novo pipeline. Initially, reads are processed to identify and count duplex tags (Make families). Reads having identical tags (families) are aligned (Align families) and alignments are reduced to DCSs (Make consensus reads). The DCSs are trimmed to remove ambiguous nucleotides (Sequence Content Trimmer), converted to fastq format (this is because DCSs are reported as fasta datasets; Combine FASTA and QUAL), and mapped to the reference genomes (in this example with both BWA and BWA-MEM). BAM datasets produced by mappers are combined (MergeSamFiles) and realigned (BamLeftAlign) and variable sites are identified with the Naive Variant Caller (NVC). A Variable Call Format (VCF) dataset generated by NVC is processed by Variant Annotator, which tabulates allele frequencies and strand bias values. Finally, the data are filtered on MAF (≥0.5 %) and strand bias (<1). This workflow is available at https://usegalaxy.org/u/aun1/w/duplex-analysis-from-reads. The most computationally demanding portion of the workflow is the alignment of reads within each family (Align Families). For instance, processing of 6,921,891 read pairs comprising the *ABL1* dataset [9] took an average of 0.004 s per pair or approximately 9 h of wall time on a 16-CPU cluster node. One of the advantages of using Galaxy at https://usegalaxy.org for the analysis of duplex sequencing data is that its underlying infrastructure relies on high-performance resources provided by the Texas Advanced Computing Center (TACC) and the Extreme Science and Engineering Discovery Environment (XSEDE), making it possible to perform analyses of multiple duplex datasets by multiple users simultaneously.

## Conclusions

The continuing drop in the price of massively parallel sequencing will expand the use of the duplex technique and will amplify the need for a scalable analysis solution such as Du Novo reported here. Our approach allows the use of both single- and double-stranded consensuses for variant discovery depending on the experimental design and is parallelized to take advantage of the advanced high performance compute infrastructure. By allowing our tools to be used both from the command line and through the Galaxy interface we hope to reach a wide audience of computational and experimental researchers.

## Methods

### Duplex sequencing protocol used for human mitochondrial amplicons

Two overlapping mtDNA regions (each ~9 kb, representing the entire mitochondrial genome) were amplified from sample SC8C1-k1169-A*B (DNA extracted from buccal swabs of the child of family SC8 collected under IRB 30432EP), using the primer pairs L*2817 + H*11570 and L10796 + H3370 and mixed at equimolar quantities, as described previously [7, 26]. Amplicons (2 μg) were sheared to ~550 bp and purified using 1.6 volumes of Agencourt AMPure XP beads (Beckman Coulter). Duplex sequencing libraries were prepared as described in Kennedy et al. [23] with several minor modifications. Briefly, T-tailed adapters were prepared by hybridization of MWS51 and MWS55, followed by extension, and a restriction digest with TaaI (HypCH4III) at 60 °C for 16 h. Adapters were purified by precipitation with two volumes of absolute ethanol and 0.5 volumes of 5 M NH_4_OAc. The hybridized PCR amplicon was end-repaired with the End-Repair Enzyme Mix provided in the Illumina TruSeq Kit according to the manufacturer’s protocol and A-tailed and the adapter was ligated with 1800 units of T4 ligase (NEB) with 20× molar excess at 16 °C for 30 min. Amplified tag families were generated from 15 attomoles of adapter-ligated amplicon by 23 cycles of PCR (the optimal cycle number was evaluated by real-time PCR). The library was quantified with the KAPA Library Quantification Kit (Kapa Biosystems) according to the manufacturer’s instructions. Sequencing was performed on an Illumina MiSeq platform using 301-bp paired-end reads.

### Construction of read families

Read pairs are grouped into families according to the random tags which constitute the first 12 bp of each read using the Du Novo pipeline either in Galaxy or through the command line. For each pair, we first construct a barcode which is the concatenation of the two tags from the two reads. Then the reads are sorted according to this compound barcode. Single-stranded families from the same fragment will have the same 12-bp tags but in the opposite order: the α tag from one family will be the β tag in the other. In order to group single-stranded families from the same fragment together, we normalize the order of the concatenation to produce a “canonical barcode” which will be identical for both strands. The order of the canonical barcode is determined by a simple string comparison. Then the original order of the tags is recorded in a separate field. Sorting the output groups the reads so that the two families constituting each duplex will be adjacent, with the read pairs separated by strand.

### Aligning families and consensus calling

The reads in each single-stranded family are aligned to themselves using a script calling the MAFFT multiple sequence aligner [27]. These alignments were used to call the SSCSs. First, a threshold is applied, requiring a specified number (default = 3) of reads to produce a consensus. Then, the consensus calling is performed by determining the majority base at each position. If no base is in the majority, “N” is used. Positions with gaps are considered in the same way as bases. Quality filtering is done at this stage: bases with a PHRED quality score lower than a user-given threshold are not counted (default = 20). For positions with gaps, a quality score is calculated by considering the quality scores of the eight nearest bases. The calculated score is a weighted average, with the weight decreasing linearly with distance from the gap. Finally, duplex consensus sequences are called using the two SSCSs. The two sequences are aligned using the Smith–Waterman algorithm (using an existing C implementation from https://code.google.com/archive/p/swalign/) and then each pair of bases is compared. If the bases agree, that base is used in that position. If they disagree, the IUPAC ambiguity code for the two bases is used. Gap and non-gap characters produce an “N”. If a SSCS has no matching opposite strand consensus, the user may choose to include the single-stranded consensus in the output, direct it to a separate file, or discard it.

### In silico mixture experiment

We randomly generated 21 heteroplasmies and inserted them into the human mitochondrial genome (Revised Cambridge Reference Sequence (rCRS), NC_012920.1) at a spacing of at least 600 bp from each other and from the chromosome ends (the genome is circular but its textual representation is not). This in silico mutated sequence is referred to as *mt-mut* to distinguish it from the unmodified reference, which we would call *mt-ref*. Next, 600-bp fragments were randomly generated using wgsim (version 0.3.1-r13) with the error and mutation rate set to 0. For mt-mut and mt-ref we generated 2500 and 25,000,000 of such fragments, respectively. Each of the fragments was tagged on each end with a random, 12-bp barcode and a 5-bp linker sequence. Each was then subjected to in silico PCR and sequencing to create a family of reads descended from the same fragment. To determine the size of the family, a random number was chosen from an empirically determined distribution, with a peak at nine reads. A phylogenetic tree was simulated for the reads by starting at the last PCR cycle and coalescing backward, randomly joining branches based on the probability of two reads sharing an ancestor at that cycle (2^-cycle^). Thirty cycles of PCR were simulated. Then, PCR polymerase errors were simulated by introducing random errors at each cycle, accumulating errors from each parent molecule. The error rate was 0.001 probability of an error per base, per cycle. Indels were given a 0.15 fraction of the errors and a 0.3 probability of extension per base. Finally, a pair of 250-bp reads was generated from each final fragment sequence. Sequencing polymerase errors were introduced at the same rates as PCR polymerase errors. Quality scores were not simulated and set to a PHRED value of 40. The strandedness of each read pair was determined according to the initial two potential daughters of the original fragment it was descended from.

Duplex consensus reads were created from these simulated reads using Du Novo with three reads required per single-stranded consensus and base quality filtering turned off (PHRED threshold of 0). The reads were aligned to the mitochondrial genome (rCRS) with BWA-MEM and filtered for alignments with a minimum mapping quality (MAPQ) of 20.

### Error rate-singleton correlation simulation

In silico duplex sequencing of the human mitochondrial reference sequence (rCRS) was performed as described above but with 10,000 400-bp fragments and 100-bp final reads to save computational time. Then, the reads were processed with the first part of the Du Novo pipeline, creating a strand-independent barcode from each read pair. Then, the total number of unique barcodes was counted and the fraction of those that were present only once. This was performed once for each error rate setting.

## Additional files

Additional file 1: Figure S1. Receiver operating characteristic (*ROC*) for Du Novo detecting 21 artificial heteroplasmies in a simulated duplex sequencing experiment. Shown are true positives versus false positives detected using different minor allele frequency thresholds, in steps of 0.00001 (the depth of coverage threshold was held constant at 10,000×). At the *bottom left*, no heteroplasmies at all are detected at a threshold MAF of 0.00016. The first variant is detected at a MAF of 0.00015, with no false positives. Continuing upward, no false positives are detected while increasing true positives are found until the *upper left corner* at a MAF of 0.00008, with 20 true positives and no false positives. Then, increasing false positives are found with no gain in true positives until the last true single-nucleotide variant (*SNV*) is found at a MAF of 0.00004, with 46 false positives also observed at that threshold. (PNG 23 kb) Additional file 2: Figure S2. Distribution of reads per family in *ABL1* (**a**) and SC8 (**b**) datasets. (JPEG 114 kb) Additional file 3: Figure S3. Here there are two distinct types of mitochondrial genomes: carrying A and G. Because the population of genomes is enriched via PCR, heteroduplex formation takes place, skewing frequency estimates performed using DCSs. If this PCR-derived DNA is now used as the starting material for a duplex sequencing experiment, the heteroduplex molecules will manifest themselves as having an N base at this site (because Du Novo interprets disagreements as Ns during consensus generation). So, DCSs produced from this dataset will have A, G, and N at the polymorphic site. Yet, SSCSs will only have A and G. Thus, SSCS will give a more accurate estimate of the allele frequency at this site in this particular case. (JPEG 196 kb) Additional file 4: Figure S4. Effect of errors on the number of single-read families. Duplex sequencing was simulated using different values for the PCR/sequencing polymerase error rates. In each case, 10,000 400-bp fragments were generated from the mitochondrial reference sequence. After simulating the duplex method, the number of reads observed for each unique barcode was counted. Shown are the fraction of families with only one read versus the polymerase error rate. (PNG 26 kb)

## Abbreviations

DCS: duplex consensus sequence
MAF: minor allele frequency
mtDNA: mitochondrial DNA
NVC: Naïve Variant Caller
PCR: polymerase chain reaction
SB: strand bias
SNP: Single nucleotide polymorphism
SSCS: single-stranded consensus sequence

## References

[1] Hodgkinson A, Idaghdour Y, Gbeha E, Grenier J-C, Hip-Ki E, Bruat V, Goulet J-P, de Malliard T, Awadalla P (2014). High-resolution genomic analysis of human mitochondrial RNA sequence variation. Science..

[2] Zhang P, Samuels DC, Lehmann B, Stricker T, Pietenpol J, Shyr Y, Guo Y (2016). Mitochondria sequence mapping strategies and practicability of mitochondria variant detection from exome and RNA sequencing data. Brief Bioinform..

[3] Schirmer M, Ijaz UZ, D’Amore R, Hall N, Sloan WT, Quince C (2015). Insight into biases and sequencing errors for amplicon sequencing with the Illumina MiSeq platform. Nucleic Acids Res..

[4] Li M, Stoneking M (2012). A new approach for detecting low-level mutations in next-generation sequence data. Genome Biol..

[5] Li M, Schröder R, Ni S, Madea B, Stoneking M (2015). Extensive tissue-related and allele-related mtDNA heteroplasmy suggests positive selection for somatic mutations. Proc Natl Acad Sci U S A..

[6] Goto H, Dickins B, Afgan E, Paul IM, Taylor J, Makova KD, Nekrutenko A (2011). Dynamics of mitochondrial heteroplasmy in three families investigated via a repeatable re-sequencing study. Genome Biol..

[7] Rebolledo Jaramillo B, Su MS-W, Stoler N, McElhoe JA, Dickins B, Blankenberg D, Korneliussen TS, Chiaromonte F, Nielsen R, Holland MM, Paul IM, Nekrutenko A, Makova KD (2014). Maternal age effect and severe germ-line bottleneck in the inheritance of human mitochondrial DNA. Proc Natl Acad Sci U S A..

[8] Quail MA, Smith M, Coupland P, Otto TD, Harris SR, Connor TR, Bertoni A, Swerdlow HP, Gu Y (2012). A tale of three next generation sequencing platforms: comparison of Ion Torrent. Pacific Biosciences and Illumina MiSeq sequencers. BMC Genomics..

[9] Ross MG, Russ C, Costello M, Hollinger A, Lennon NJ, Hegarty R, Nusbaum C, Jaffe DB (2013). Characterizing and measuring bias in sequence data. Genome Biol.

[10] Wallace DC, Chalkia D (2013). Mitochondrial DNA genetics and the heteroplasmy conundrum in evolution and disease. Cold Spring Harb Perspect Biol..

[11] Dimond R (2015). Social and ethical issues in mitochondrial donation. Br Med Bull..

[12] Jabara CB, Jones CD, Roach J, Anderson JA, Swanstrom R (2011). Accurate sampling and deep sequencing of the HIV-1 protease gene using a Primer ID. Proc Natl Acad Sci U S A..

[13] Schmitt MW, Fox EJ, Prindle MJ, Reid-Bayliss KS, True LD, Radich JP, Loeb LA (2015). Sequencing small genomic targets with high efficiency and extreme accuracy. Nat Methods..

[14] Kim SY, Lohmueller KE, Albrechtsen A, Li Y, Korneliussen T, Tian G, Grarup N, Jiang T, Andersen G, Witte D, Jorgensen T, Hansen T, Pedersen O, Wang J, Nielsen R (2011). Estimation of allele frequency and association mapping using next-generation sequencing data. BMC Bioinformatics..

[15] Hindson CM, Chevillet JR, Briggs HA, Gallichotte EN, Ruf IK, Hindson BJ, Vessella RL, Tewari M (2013). Absolute quantification by droplet digital PCR versus analog real-time PCR. Nat Methods..

[16] Miotke L, Lau BT, Rumma RT, Ji HP (2014). High sensitivity detection and quantitation of DNA copy number and single nucleotide variants with single color droplet digital PCR. Anal Chem.

[17] Schmitt MW, Kennedy SR, Salk JJ, Fox EJ, Hiatt JB, Loeb LA (2012). Detection of ultra-rare mutations by next-generation sequencing. Proc Natl Acad Sci U S A..

[18] Ewing B, Green P (1998). Base-calling of automated sequencer traces using phred. II. Error probabilities. Genome Res..

[19] Smith TF, Waterman MS (1981). Identification of common molecular subsequences. J Mol Biol..

[20] Blankenberg D, Von Kuster G, Bouvier E, Baker D, Afgan E, Stoler N, Taylor J, Nekrutenko A, Galaxy Team (2014). Dissemination of scientific software with Galaxy ToolShed. Genome Biol.

[21] Garrison E, Marth G. Haplotype-based variant detection from short-read sequencing. arXiv:1207.3907 arXiv.org 2012.

[22] Guo Y, Li J, Li C-I, Long J, Samuels DC, Shyr Y (2012). The effect of strand bias in Illumina short-read sequencing data. BMC Genomics..

[23] Kennedy SR, Schmitt MW, Fox EJ, Kohrn BF, Salk JJ, Ahn EH, Prindle MJ, Kuong KJ, Shen J-C, Risques R-A, Loeb LA (2014). Detecting ultralow-frequency mutations by Duplex Sequencing. Nat Protoc..

[24] Li H. Aligning sequence reads, clone sequences and assembly contigs with BWA-MEM. arXiv:1303.3997 arXiv.org 2013.

[25] Li H, Durbin R. Fast and accurate short read alignment with Burrows-Wheeler Transform. Bioinformatics. 2009;1–7. 10.1093/bioinformatics/btp324 PMC2705234 19451168

[26] Dickins B, Rebolledo Jaramillo B, Su MS-W, Paul IM, Blankenberg D, Stoler N, Makova KD, Nekrutenko A (2014). Controlling for contamination in re-sequencing studies with a reproducible web-based phylogenetic approach. Biotechniques..

[27] Katoh K, Standley DM (2013). MAFFT multiple sequence alignment software version 7: improvements in performance and usability. Mol Biol Evol..

