# Supplementary figures and images for: Streamlined analysis of duplex sequencing data with Du Novo
Source: Genome Biol. 2016 Aug 26;17(1):180. doi: 10.1186/s13059-016-1039-4 (PMC5000403; doi:10.1186/s13059-016-1039-4)

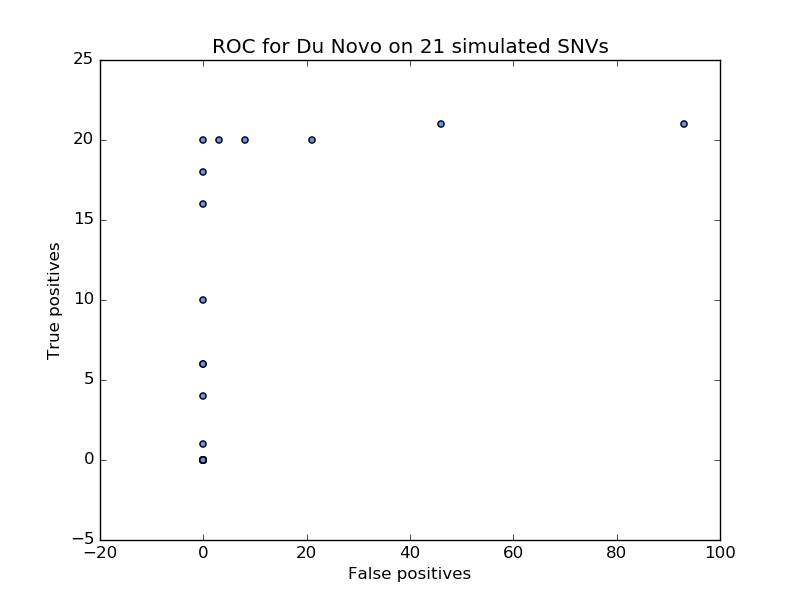

Supplement: Additional file 1: Figure S1. — Receiver operating characteristic (ROC) for Du Novo detecting 21 artificial heteroplasmies in a simulated duplex sequencing experiment. Shown are true positives versus false positives detected using different minor allele frequency thresholds, in steps of 0.00001 (the depth of coverage threshold was held constant at 10,000×). At the bottom left, no heteroplasmies at all are detected at a threshold MAF of 0.00016. The first variant is detected at a MAF of 0.00015, with no false positives. Continuing upward, no false positives are detected while increasing true positives are found until the upper left corner at a MAF of 0.00008, with 20 true positives and no false positives. Then, increasing false positives are found with no gain in true positives until the last true single-nucleotide variant (SNV) is found at a MAF of 0.00004, with 46 false positives also observed at that threshold. (PNG 23 kb) [file 13059_2016_1039_MOESM1_ESM.png]

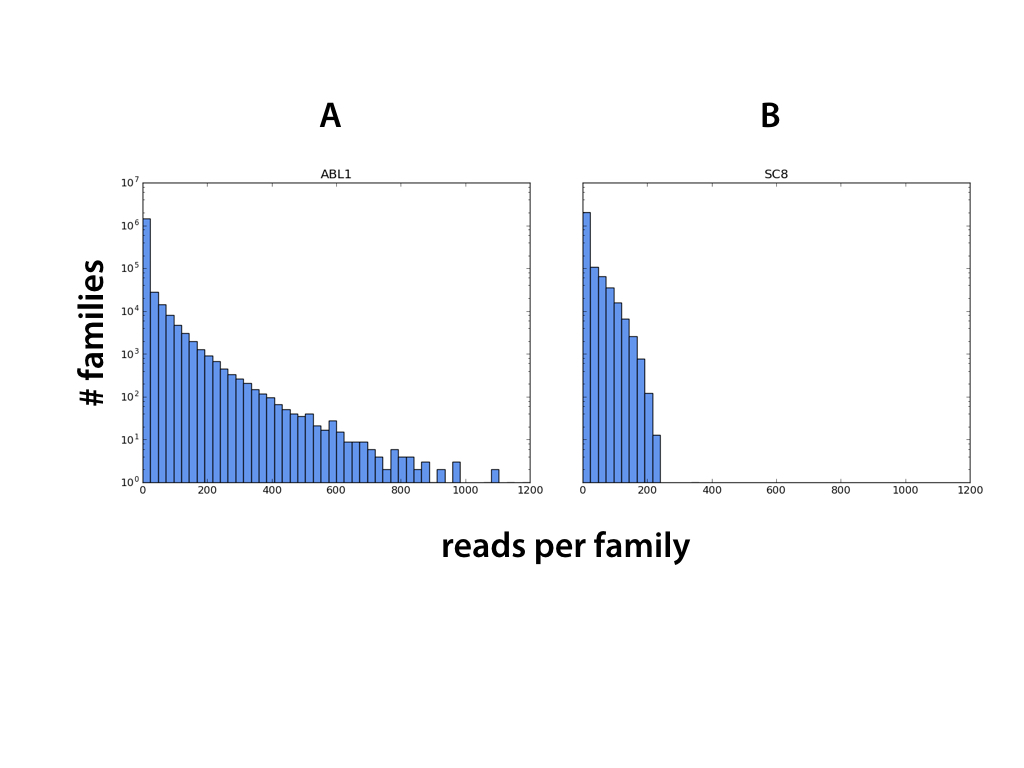

Supplement: Additional file 2: Figure S2. — Distribution of reads per family in ABL1 (a) and SC8 (b) datasets. (JPEG 114 kb) [file 13059_2016_1039_MOESM2_ESM.jpeg]

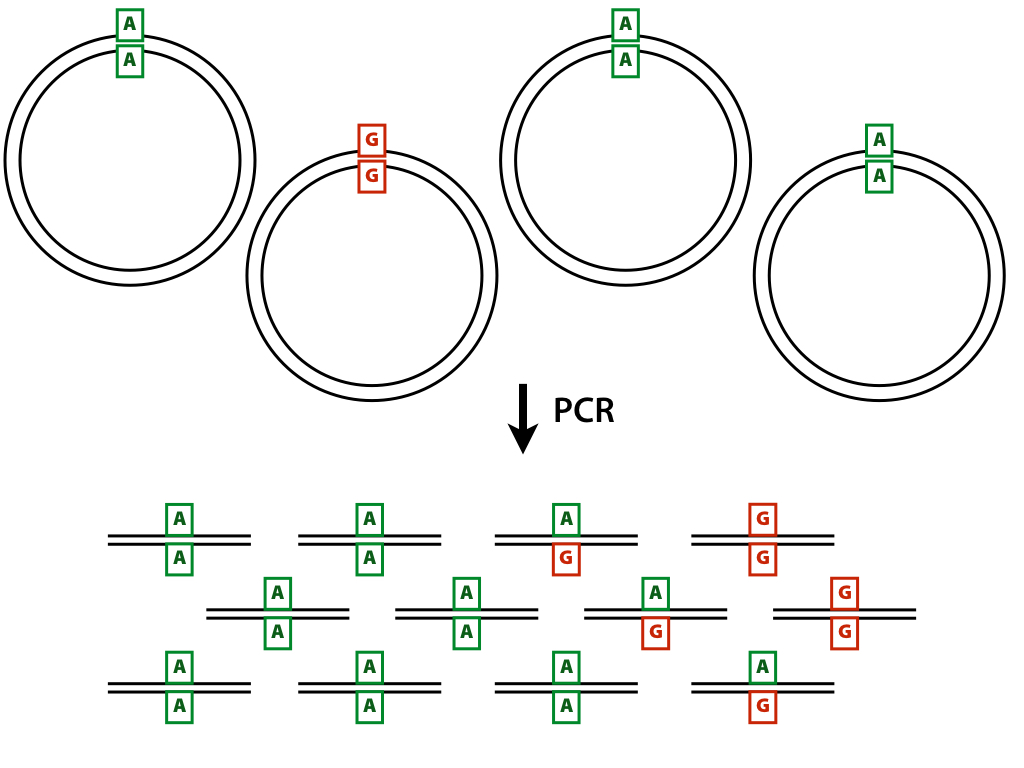

Supplement: Additional file 3: Figure S3. — Here there are two distinct types of mitochondrial genomes: carrying A and G. Because the population of genomes is enriched via PCR, heteroduplex formation takes place, skewing frequency estimates performed using DCSs. If this PCR-derived DNA is now used as the starting material for a duplex sequencing experiment, the heteroduplex molecules will manifest themselves as having an N base at this site (because Du Novo interprets disagreements as Ns during consensus generation). So, DCSs produced from this dataset will have A, G, and N at the polymorphic site. Yet, SSCSs will only have A and G. Thus, SSCS will give a more accurate estimate of the allele frequency at this site in this particular case. (JPEG 196 kb) [file 13059_2016_1039_MOESM3_ESM.jpeg]

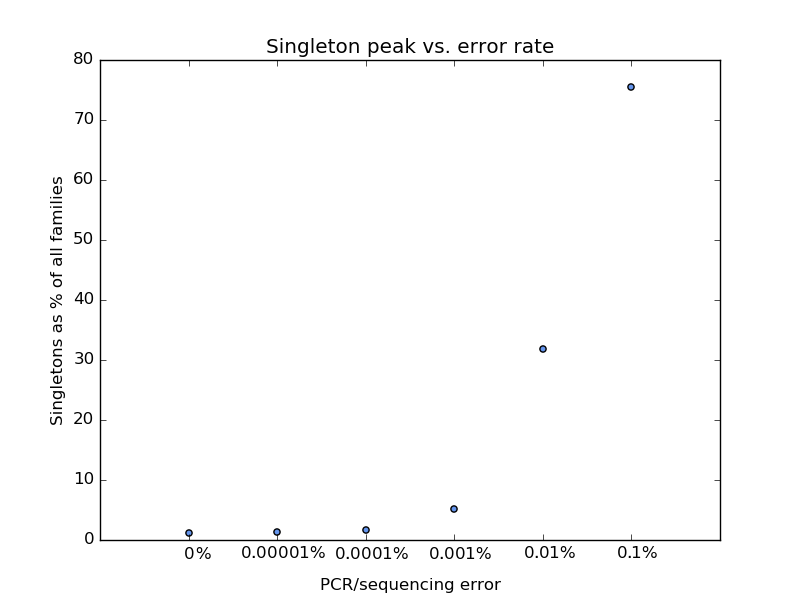

Supplement: Additional file 4: Figure S4. — Effect of errors on the number of single-read families. Duplex sequencing was simulated using different values for the PCR/sequencing polymerase error rates. In each case, 10,000 400-bp fragments were generated from the mitochondrial reference sequence. After simulating the duplex method, the number of reads observed for each unique barcode was counted. Shown are the fraction of families with only one read versus the polymerase error rate. (PNG 26 kb) [file 13059_2016_1039_MOESM4_ESM.png]
